# Slow but Steady—The Responsiveness of Sympathoadrenal System to a Hypoglycemic Challenge in Ketogenic Diet-Fed Rats

**DOI:** 10.3390/nu13082627

**Published:** 2021-07-29

**Authors:** Polina E. Nedoboy, Myfanwy Cohen, Melissa M.-J. Farnham

**Affiliations:** 1 The Heart Research Institute, Newtown, NSW 2042, Australia; polina.nedoboy@hri.org.au (P.E.N.); myfanwy_cohen@hotmail.com (M.C.); 2 Sydney Medical School, The University of Sydney, Sydney, NSW 2006, Australia

**Keywords:** counterregulatory response, epinephrine, insulin-induced hypoglycemia, ketosis, ketogenic diet, rat model

## Abstract

The sympathoadrenal counterregulatory response to hypoglycemia is critical for individuals with type 1 diabetes due to impaired ability to produce glucagon. Ketogenic diets (KD) are an increasingly popular diabetes management tool; however, the effects of KD on the sympathoadrenal response are largely unknown. Here, we determined the effects of KD-induced ketosis on the sympathoadrenal response to a single insulin-induced hypoglycemic challenge. We investigated how a 3 week KD feeding regimen affected the main components of the sympathoadrenal counterregulatory response: adrenal sympathetic nerve activity (ASNA), adrenal gland activity, plasma epinephrine, and brainstem glucose-responsive C1 neuronal activation in anesthetized, nondiabetic male Sprague-Dawley rats. Rats on KD had similar blood glucose (BG) levels and elevated ketone body β-hydroxybutyrate (BHB) levels compared to the control Chow diet group. All KD rats responded to hypoglycemia with a robust increase in ASNA, which was initiated at significantly lower BG levels compared to Chow-fed rats. The delay in hypoglycemia-induced ASNA increase was concurrent with rapid disappearance of BHB from cerebral and peripheral circulation. Adrenal gland activity paralleled epinephrine and ASNA response. Overall, KD-induced ketosis was associated with initiation of the sympathoadrenal response at lower blood glucose levels; however, the magnitude of the response was not diminished.

## 1. Introduction

Insulin therapy is the gold standard of treatment for type 1 diabetes and patients with advanced type 2 diabetes, but maintaining consistent blood glucose levels is challenging. Hypoglycemia frequently results from inadvertent excessive administration of insulin [1] and, if untreated, can lead to serious neurological consequences, such as mental confusion, seizures, and coma. Severe hypoglycemia is a major obstacle to optimal management of type 1 diabetes with insulin and it is not just debilitating, it can be fatal, accounting for up to 10% of deaths in this population [2].

In individuals without diabetes, hypoglycemia is initially counteracted by increased glucagon secretion and suppression of endogenous insulin release [3]. If blood glucose levels continue to fall (<3.8 mmol/L), a highly coordinated neural counterregulatory reflex is triggered which activates the sympathoadrenal system [4]. This reflex response is initiated by falling extracellular glucose, which activates a subset of glucose-sensing neurons in the hypothalamus and brainstem, termed glucose-inhibited neurons. Activated glucose-inhibited neurons convey signals via sympathetic preganglionic neurons in the spinal cord, through the adrenal sympathetic nerve to adrenal chromaffin cells. Activation of epinephrine-producing chromaffin cells results in rapid release of epinephrine into the circulation [5]. This triggers physical symptoms which prompts a person to ingest carbohydrates [6], and the mobilization of glucose via glycogenolysis, gluconeogenesis, pancreatic glucagon secretion, reduced glucose uptake, and increased lipolysis (reviewed in [7]).

Unlike the nondiabetic population, type 1 diabetes patients rely almost exclusively on stimulated release of adrenal epinephrine to raise blood glucose, due to severely compromised pancreatic glucagon release in response to insulin-induced hypoglycemia [8] and an inability to stop the action of already injected insulin. A failure to mount an adequate sympathoadrenal response can compromise a patient’s ability to both physically sense and behaviorally respond to an episode of hypoglycemia, putting them at risk of seizures, coma, and death—the most severe manifestations of neuroglycopenia (cerebral glucose deprivation). 

Under normal physiological conditions, glucose is the only readily available energy source for the brain. During prolonged fasting, ketone bodies (acetoacetate, β-hydroxybutyrate (BHB) and acetone) which are small molecules produced from fatty-acid oxidation in the liver, can readily cross the blood brain barrier and provide an additional energy source [9]. Ketosis has neuroprotective effects against hypoglycemia in both type 1 diabetes patients [10,11] and rats [12]. Physiological levels of circulating ketones are low, 0–0.5 mmol/L; however, during extended fasting, ketones can accumulate to 7–8 mmol/L [13] and provide up to two-thirds of the brain’s energy requirements [14]. A carbohydrate-restricted diet, called the ketogenic diet (KD), also induces a state of stable ketosis after a period of adaptation, which is comparable to extended fasting conditions [15]. The KD (55–60% fat, 30–35% protein, and 5–10% carbohydrate) has been used for almost a century for the treatment of drug-resistant epilepsy in children [16] due to its anticonvulsant properties; in recent years, the KD has also become an increasingly popular nutritional strategy for people with type 1 diabetes, owing to significant decreases in insulin requirements and greater blood glucose stability between meals [17]. However, there are reports concerning an increased number of non-symptomatic hypoglycemia cases, recorded with the aid of continuous glucose monitors [18,19], possibly due to a shift of the glycemic threshold for sympathoadrenal/sympathoneural symptoms to lower blood glucose levels [18]. To date, no studies have investigated the effects of nutritional ketosis, induced by the KD, on the sympathoadrenal counterregulatory response (CRR) to hypoglycemia. In contrast, a number of studies concerning the effects of acute bolus administration of exogenous ketones on various components of the hypoglycemia CRR in humans have been reported, with conflicting results; ingestion of ketone precursors (medium chain triglycerides) by intensively treated type 1 diabetic patients did not decrease the levels of counterregulatory hormones in response to insulin-induced hypoglycemia [10], and it did not diminish in healthy humans upon BHB infusion in one study [20], yet, in another study, acute administration of BHB in healthy humans significantly reduced the CRR [11].

Previous studies in KD-fed nondiabetic mice reported an impairment in glucagon secretion in response to insulin-induced hypoglycemia [21]. Although acute administration of glucagon to hypoglycemic type 1 diabetes patients is an effective treatment to stimulate endogenous glucose production, its role in the endogenous CRR in type 1 diabetes is limited due to pancreatic alpha- and beta-cell failure. For that reason, and the fact that glucagon injections are less effective in patients who follow a low-carbohydrate diet [22], our study focused specifically on the role of the sympathoadrenal system and epinephrine, which are expected to be the major contributors to the CRR in the setting of type 1 diabetes. Therefore, the aim of this study was to test the effects of dietary ketosis, induced by a 3 week KD, on components of the sympathoadrenal CRR to severe insulin-induced hypoglycemia in healthy, nondiabetic rats, by measuring adrenal sympathetic nerve activity (ASNA), adrenal gland activity, and epinephrine release, as well as the activation state of catecholaminergic C1 neurons in the brainstem, which are glucose-sensitive and involved in the hypoglycemic CRR [23,24]. The adrenal sympathetic nerve, unlike renal, lumbar, or muscle sympathetic nerves, is activated by hypoglycemia independent of elevated insulin levels [25] and can be acutely recorded in anesthetized rats [26]. As ASNA positively correlates with epinephrine release [27], and the actions of epinephrine on target tissues produces physical symptoms of hypoglycemia, in vivo ASNA recordings in anesthetized rats can provide a sensitive, real-time measure of hypoglycemia progression without confounders such as handling stress, multiple blood draws for plasma epinephrine quantification, and difficulties in registering mild physical symptoms in conscious animals. We hypothesize that the CRR is likely to be reduced, and the time course of its initiation may be shifted, as the KD may provide additional substrate and spare glucose for neuronal metabolism in the face of diminishing glucose availability, thereby delaying the central sympathoadrenal response.

## 2. Materials and Methods

### 2.1. Animals

All experiments were conducted in accordance with the Australian Code of Practice for the Care and Use of Animals for Scientific Purposes (New South Wales: Animal Re-search Act 1985) and approved by the Sydney Local Health District Animal Welfare Committee (AWC #2018/014). Adult male Sprague-Dawley rats (325–485 g, *n* = 27) were fed either a ketogenic diet (KD, SF10-053, 69% fat, 16% protein, 1.2% digestible carbohydrate, Ketogenic Rodent Diet, Specialty Feeds, Glen Forrest, WA, Australia, *n* = 12) or a control standard irradiated rodent diet (Chow, 4.6% fat, 19% protein, 59.9% total carbohydrate, Specialty Feeds, Glen Forrest, WA, Australia, *n* = 15) ad libitum with free access to drinking water for 3 weeks. Rats were housed (3 per cage) at the Heart Research Institute animal facility at 23–25 °C, 12 h dark/light cycles, 50–60% humidity. Following dietary manipulation, rats were randomly divided into insulin or PBS groups: Chow + insulin (*n* = 12), Chow + PBS (*n* = 3), KD + insulin (*n* = 9), or KD + PBS (*n* = 3).

### 2.2. Anesthesia

Sodium pentobarbital (Virbac, Milperra, NSW, Australia) has minimal effects on blood glucose levels and Fos expression [28,29] and, therefore, was used for anesthesia in overnight fasted rats (induction: 65 mg/kg ip; maintenance: 13 mg/h iv). The depth of anesthesia was monitored by checking blood pressure responses to tail or paw pinches and was adjusted if changes exceeded 10 mmHg. Arterial PO_2_, PCO_2_, and pH were kept within the physiological range by monitoring end-tidal CO_2_ throughout the experiment and adjusting ventilatory rate and volume when necessary. Core body temperature was maintained at 36.5–37.5 °C.

### 2.3. Cannulations and Nerve Recordings

The right carotid artery and jugular vein were cannulated (for the recording of arterial blood pressure and administration of drugs, respectively), and a tracheostomy was per-formed for mechanical ventilation and recording of end-tidal CO_2_. Animals were ventilated with room air and supplemental 100% O_2_. Heart rate was derived from a three-lead ECG. The left adrenal sympathetic nerve was isolated and carefully placed on silver bipolar electrode as previously described [26]. Neurograms were sampled at 2000 Hz, bandpass filtered (10 Hz–3 kHz), and amplified 5000 times (BMA-931 Bioamplifier, CWE, Ardmore, PA, USA). As the adrenal sympathetic nerve can contain a mixture of preganglionic (activated by glucoprivation and synapsing on catecholaminergic chromaffin cells) and postganglionic nerve fibers (innervating the adrenal cortex), upon completion of the 2 h recording period, a ganglion blocker hexamethonium (40 mg/kg, Sigma-Aldrich, St. Louis, MO, USA) was administered intravenously. Experiments (*n* = 2, KD + Ins group) were excluded from analysis of adrenal sympathetic nerve activity (ASNA) and all related analyses as the ratio of pre-to-postganglionic activity was less than 50% [27]. As such, these recorded nerves were not predominantly preganglionic and did not reflect the specific hypoglycemia-induced changes in activity; therefore, they were deemed sub-optimal. ASNA recordings were rectified, smoothed (τ = 2 s), and normalized to baseline (100% activity) by subtracting residual activity (0%) after crushing the nerve with forceps at the conclusion of experiment. Mean ASNA, expressed as a percentage change from baseline, was analyzed in 3 min intervals taken immediately before insulin administration (0 min) and 15, 30, 45, 60, 90, and 120 min post insulin administration. 

### 2.4. Insulin Injections

In unpublished pilot studies conducted in our laboratory, we established that a single intravenous injection of 5 U/kg of insulin in anesthetized, overnight fasted rats produced a consistent and highly reproducible level of hypoglycemia in both Chow- and KD-fed groups. Such a consistent degree of hypoglycemia allowed tracking the changes in ASNA with changes in blood glucose levels over time without the need for a hypoglycemic-hyperinsulinemic clamp. In the current study, hypoglycemia was induced in the Chow-fed (*n* = 9) and KD-fed (*n* = 7) rats by a single intravenous injection of insulin (human recombinant, 5 U/kg, I2643, Sigma-Aldrich, North Ryde, NSW, Australia). The control Chow (*n* = 3) and KD-fed (*n* = 3) rats were injected the same volume of PBS. Peripheral blood glucose and BHB were measured from a drop of blood, obtained via a tail nick, at 0 (pre insulin administration), 15, 30, 45, 60, 90, and 120 min. Baseline (0 min, pre insulin administration) blood glucose and BHB were consistently measured between 11:00 a.m. and 12:00 p.m. An Accu-check Performa glucose meter (Roche Diabetes Care, North Ryde, NSW, Australia) and corresponding glucose strips were used for glucose determination, and an Abbott Optium Neo glucose and ketone meter with Freestyle Optium ketone strips (Abbott Diabetes Care, Doncaster, VIC, Australia) were used for BHB measurements. 

### 2.5. Measurement of Cerebral Venous BHB Levels

To confirm the presence and utilization of BHB in the brain, cerebral venous blood BHB was sampled in a small separate group of animals (KD + Ins *n* = 2 and Chow + Ins *n* = 3) at baseline (0 min) and 15, 30, 45, 60, 90, and 120 min post insulin injection. The skull was exposed, and a 4–5 mm diameter hole was drilled above the confluence of sinuses posterior and central to Lambda (Supplementary Figure S1). The confluence of sinuses was opened by puncturing the dura with a needle. Venous blood was sampled at specified times with the ketone strips as described above, by reopening the hole in the confluence of sinuses and letting it bleed for ~7 s before occluding it with gauze.

### 2.6. Epinephrine ELISA

At the conclusion of the recording period, blood was collected via the carotid artery catheter. Plasma was separated, aliquoted, and stored at −80 °C. Ultra-sensitive epinephrine ELISA (KA3837, Abnova, Taipei, Taiwan) was performed according to the manufacturer’s instructions. 

### 2.7. Immunohistochemistry

Two hours post insulin administration and following blood collection, rats were transcardially perfused with ice-cold PBS and 4% paraformaldehyde. Adrenal glands and the brain were removed and post-fixed for 24 h. Fixed adrenal glands (3–5 per treatment group) were embedded together in 2% agar and sectioned on a vibrating microtome at 20 µm in 1:5 series. Brainstem (*n* = 3 per treatment group) was sectioned coronally at 40 µm in 1:5 series. Free-floating sections underwent routine immunohistochemistry [24]. Primary antibodies for adrenal gland immunohistochemistry were anti-PNMT (phenylethanolamine-*N*-methyltransferase, a marker for epinephrine-producing cells [30]; 1:1000, rabbit polyclonal, generated and characterized by P.R. Howe [31]) and anti-Fos (a marker for recently activated cells of neuronal origin; 1:1000, guinea pig polyclonal 226004; Synaptic Systems, Goettingen, Germany). Anti-tyrosine hydroxylase primary antibody (TH, a marker for catecholaminergic neurons, 1:100, mouse monoclonal, Avanti Antibodies, #AV1, previously characterized by Nedoboy et al. [32]) together with anti-Fos antibody (as above) was used for brainstem immunohistochemistry. Secondary antibodies conjugated with fluorescent tags were used to enable visualization of colocalizations. The secondary antibodies used were donkey anti-rabbit AlexaFluor488 (1:500, 711-546-152, Jackson Immunoresearch, West Grove, PA, USA) and donkey anti-guinea pig Cy5-conjugated (1:500, 706-175-148, Jackson Immunoresearch, West Grove, PA, USA) (adrenal gland), and goat anti-mouse IgG1k-specific Cy5 (1:500, 115-175-205, Jackson Immunoresearch, West Grove, PA, USA) and donkey anti-guinea pig Cy3-conjugated (1:500, 706-166-148, Jackson Immunoresearch, West Grove, PA, USA) for the brainstem. Sections were mounted on glass slides with ProLong Diamond antifade (Invitrogen, Carlsbad, CA, USA).

### 2.8. Image Acquisition and Analysis

Adrenal medullae (randomly selected five sections per treatment group) were im-aged (Zeiss Axio Imager Z2; 20×) and analyzed in Fiji ImageJ software. The thresholded Fos-positive area, measured in pixels, was normalized to the PNMT-positive area and ex-pressed as a percentage of PNMT-positive area. Fos-positive staining outside of the PNMT-immunoreactive area was excluded. Four 40 µm brainstem sections containing the C1 catecholaminergic nucleus (bregma level −12.48 to −12.12) per animal were used to count the number of TH immunoreactive cells (TH^+^), Fos immunoreactive cells (Fos^+^), and double-labeled (TH^+^/Fos^+^) cells bilaterally within the region. A rectangular region of interest (ROI, 1680 × 810 µm) was superimposed on the images taken with Zeiss Axio Imager Z2, 10× objective, and cells were counted manually within the ROI by an operator blinded to the experimental conditions.

### 2.9. Statistical Analysis

**Figure 1:**
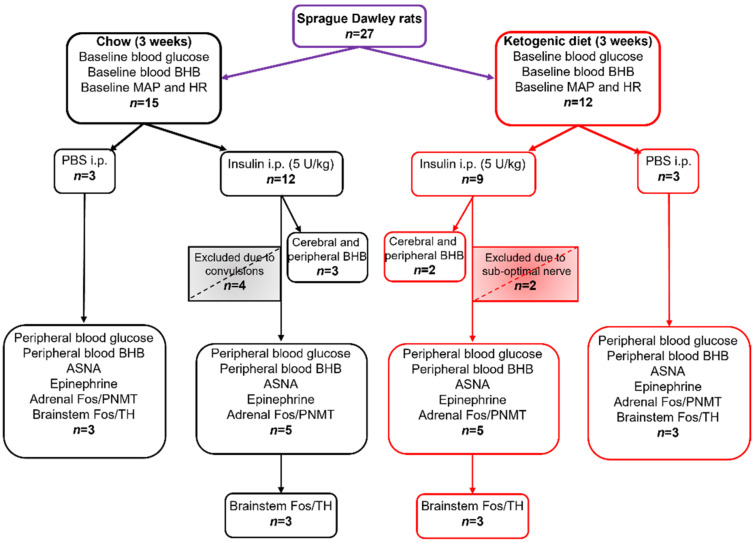
Sample sizes for dietary groups, treatments, and measured variables. MAP—mean arterial pressure; HR—heart rate; BHB—β-hydroxybutyrate; ASNA—adrenal sympathetic nerve activity, PNMT—phenylethanolamine N-methyltransferase; TH—tyrosine hydroxylase; Fos—protein product of immediate early gene *c-fos*.

All statistical analyses were performed using GraphPad Prism software (version 9.0.0). Comparisons between groups were made with unpaired *t*-test or Mann-Whitney test for non-normally distributed residuals, and one or two-way ANOVA with Holm-Šidák’s post-tests for multiple comparisons where appropriate; for data with unequal SDs, Brown-Forsythe ANOVA test was used. Results are reported as means ± SEM unless stated otherwise, with statistical significance set at *p* < 0.05. The smallest effect size was derived for ASNA, epinephrine, PNMT^+^/Fos^+^, and TH^+^/Fos^+^ measurements from the data generated from the Chow + Ins group which was considered a normal physiological response to insulin injection. To detect a difference of >20%, a minimum of three animals per group was required to achieve the power >80% for all measured variables (ASNA, epinephrine, blood glucose, BHB and Fos). Sample sizes for all treatment groups and measured variables are outlined in Figure 1.

## 3. Results

### 3.1. The Effects of KD on Blood Glucose, BHB, Arterial Pressure, and Heart Rate

**Figure 2:**
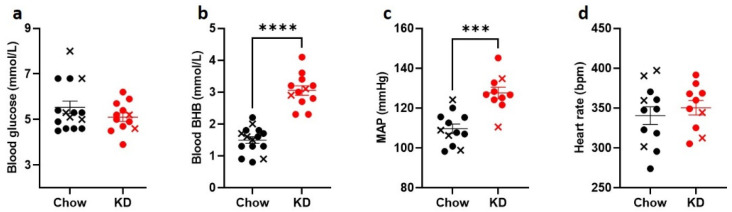
Ketogenic diet increases BHB but does not change blood glucose levels. Sprague-Dawley rats were fed ketogenic diet (KD, *n* = 10, red circles) or control diet (Chow, *n* = 12, black circles) for 3 weeks. KD-fed rats had comparable (**a**) blood glucose levels and significantly higher (**b**) blood BHB levels. (**c**), KD-fed rats had significantly elevated baseline mean arterial pressure (MAP), but not (**d**) heart rate. Crosses indicate animals that were excluded from further analysis due to hypoglycemia-induced convulsions (black) or suboptimal nerve recordings (red). Data are means ± SEM, Mann-Whitney test (**a**) and unpaired *t*-test (**b**–**d**); *** *p* < 0.001, **** *p* < 0.0001.

To induce the state of stable ketosis [33], we used a high-fat, adequate-protein, low-carbohydrate diet in male Sprague-Dawley rats. As expected, following 3 weeks of a keto-genic diet, (KD)-fed rats (*n* = 12) had significantly increased fasting BHB levels (KD: 3.1 ± 0.1 mmol/L vs. Chow: 1.5 ± 0.1 mmol/L, respectively, *p* < 0.0001, Figure 2b) but similar fasting blood glucose levels to Chow-fed rats (*n* = 15) (KD: 5.1 ± 0.2 mmol/L vs. 5.5 ± 0.3 mmol/L, *p* = 0.46, Figure 2a). Interestingly, baseline mean arterial pressure (MAP) in anesthetized animals was higher in the KD-fed rats compared to the Chow-fed animals (127 ± 3 mmHg vs. 110 ± 2 mmHg, *p* = 0.0001, Figure 2c), whereas heart rate did not differ significantly (Figure 2d).

### 3.2. Insulin Administration Lowers Blood Glucose and BHB

**Figure 3:**
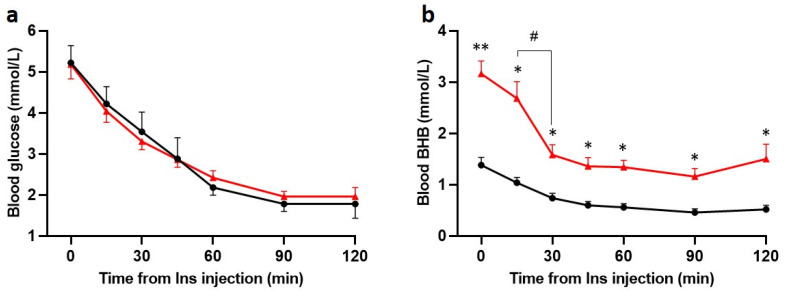
Insulin induces hypoglycemia in Chow and KD-fed rats and decreases BHB in KD-fed rats (*n* = 5 for both). Hypoglycemia was induced with intravenous injection of insulin (Ins, 5 U/kg). (**a**), Blood glucose and (**b**) BHB levels were measured at 0, 15, 30, 45, 60, 90, and 120 min post insulin administration. No significant differences (**a**, *n* = 5 both groups) in blood glucose levels were found between groups at any timepoint. (**b**), BHB levels were significantly higher in KD + Ins rats (red line, *n* = 5) at every timepoint compared to Chow + Ins rats (black line, *n* = 5); BHB sharply declined within 30 min of insulin injection in KD + Ins rats (red line). Data are means ± SEM, */# *p* < 0.05, ** *p* < 0.01, two-way ANOVA with Holm-Šidák’s post hoc tests for multiple comparisons.

Insulin (5 U/kg) consistently induced a progressive decrease in blood glucose levels in both Chow and KD groups (Figure 3a). In the KD-fed rats, BHB fell significantly within the first 30 min of insulin injection but remained considerably higher than the Chow-fed rats at every timepoint (Figure 3b). Within 120 min, BHB decreased from 3.2 ± 0.3 mmol/L to 1.5 ± 0.3 mmol/L, levels similar to the pre-insulin levels in the Chow-fed rats. The BHB levels following insulin in Chow-fed rats were physiological for fasting conditions [34]. These data indicate that both glucose and ketones are consumed following administration of insulin. Within 60 min of insulin administration, MAP decreased significantly in both Chow-fed and KD-fed rats and was no longer different between the groups (Supplementary Figure S2).

**Figure 4:**
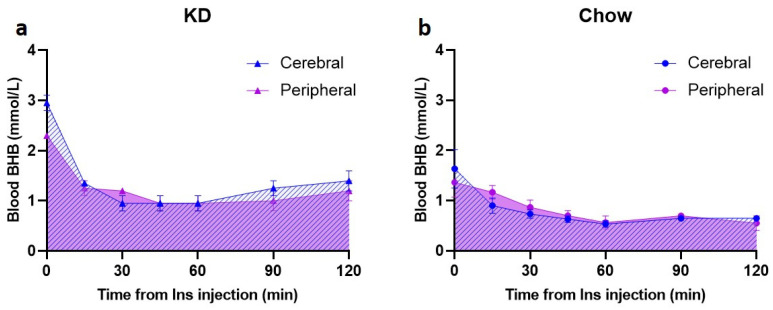
Disappearance of BHB from cerebral venous blood is rapid in the KD-fed rats. Following insulin injection, cerebral venous BHB (**a**,**b**, blue hatched area) was measured after 15, 30, 45, 60, 90, and 120 min in the blood obtained from confluence of sinuses simultaneously with peripheral blood (tail vein) BHB measurements (**a**,**b**, purple shaded area). (**a**) KD-fed animals (*n* = 2); (**b**) Chow-fed animals (*n* = 3). KD—ketogenic diet; Chow—standard rodent diet.

To assess the presence and utilization of BHB in the brain, a preliminary subset of animals (KD *n* = 2 and Chow *n* = 3) was used to measure BHB levels in the cerebral venous blood obtained from the confluence of sinuses (Supplementary Figure S1), concurrently with the peripheral blood BHB measurements. In this subgroup, there was a rapid decrease in cerebral BHB levels in KD-fed animals in the first 30 min (from 3.0 ± 0.2 mmol/L to 1.0 ± 0.2 mmol/L, Figure 4a); this change was larger than the peripheral blood BHB decrease within the same time period (2.3 ± 0.0 mmol/L to 1.2 ± 0.0 mmol/L, Figure 4a). Baseline BHB levels in the Chow-fed rats were substantially lower in both cerebral and peripheral blood (1.6 ± 0.4 mmol/L and 1.4 ± 0.3 mmol/L, respectively) compared to the KD-fed rats, and the hypoglycemia-induced decrease was modest, reaching the lowest of 0.5 mmol/L at 60 min post insulin injection (Figure 4b). In comparison, BHB nadir in the KD-fed animals was 1 mmol/L (Figure 4a).

### 3.3. KD-Induced Ketosis Shifts the Initiation of ASNA to Lower Blood Glucose Levels but Preserves the Maximal Response

**Figure 5:**
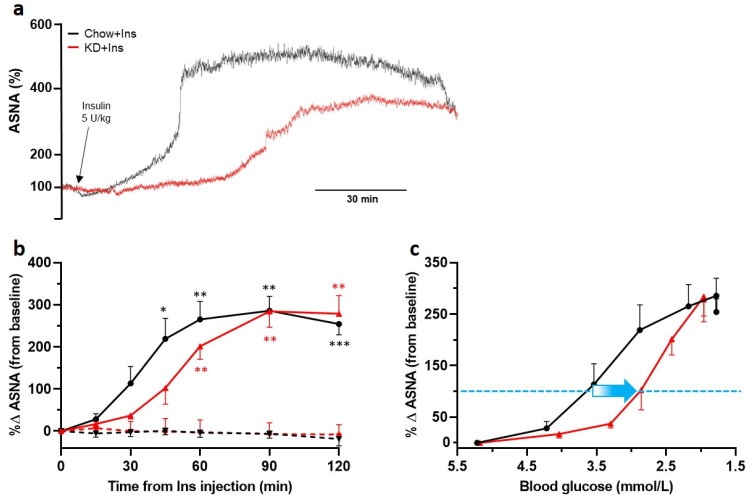
Ketogenic diet does not decrease adrenal sympathetic nerve response to hypoglycemia but shifts its initiation to lower blood glucose levels. The ASNA response was analyzed in 3 min intervals taken immediately before insulin administration (0 min) and 15, 30, 45, 60, 90, and 120 min post insulin administration. (**a**) Example traces of ASNA recorded over 120 min post insulin administration (black arrow) from KD + Ins rats (red) and Chow + Ins rats (black). (**b**) Grouped data showing significant increases in ASNA in both KD + Ins (solid red line, *n* = 5) and Chow + Ins rats (solid black line, *n* = 5), compared to the respective PBS controls (dashed red and black lines, *n* = 3 for both). (**c**) Grouped data showing a shift (blue arrow) in ASNA response to lower blood glucose levels in KD + Ins rats (red line) compared to Chow + Ins rats (black line). The dashed blue line in (**c**) indicates 100% increase in ASNA from baseline. Data are means ± SEM; * *p* < 0.05, ** *p* < 0.01, *** *p* < 0.001; two-way ANOVA with Holm-Šidák’s post hoc tests for multiple comparisons. ASNA—adrenal sympathetic nerve activity.

Insulin-induced hypoglycemia initiated a robust increase in ASNA (Figure 5a—sample traces and Figure 5b—grouped data) in all animals compared to the PBS-treated controls, indicative of a functional sympathetic CRR. Four out of nine insulin treated Chow-fed rats were not included in further analysis due to the development of clonic convulsions (presumably neuroglycopenic seizures), terminatable by glucose administration, within 2 h of recording (grouped data shown in Supplementary Table S1). None of the KD-fed rats showed any signs of clonic convulsions. The blood glucose level required to induce a doubling in ASNA (Figure 5c dashed blue line) was much lower (~2.8 mmol/L) in KD-fed than in Chow-fed rats (~3.7 mmol/L; Figure 5c). Following an initial delay, the ASNA response in KD-fed rats exponentially increased reaching a similar (if not higher) maximum to Chow-fed rats (KD 284.2 ± 38% (*n* = 5); Chow 285.6 ± 34% (*n* = 5); Figure 5b).

### 3.4. Insulin-Induced Hypoglycemia Increases the Activation of Medullary C1 Neurons

**Figure 6:**
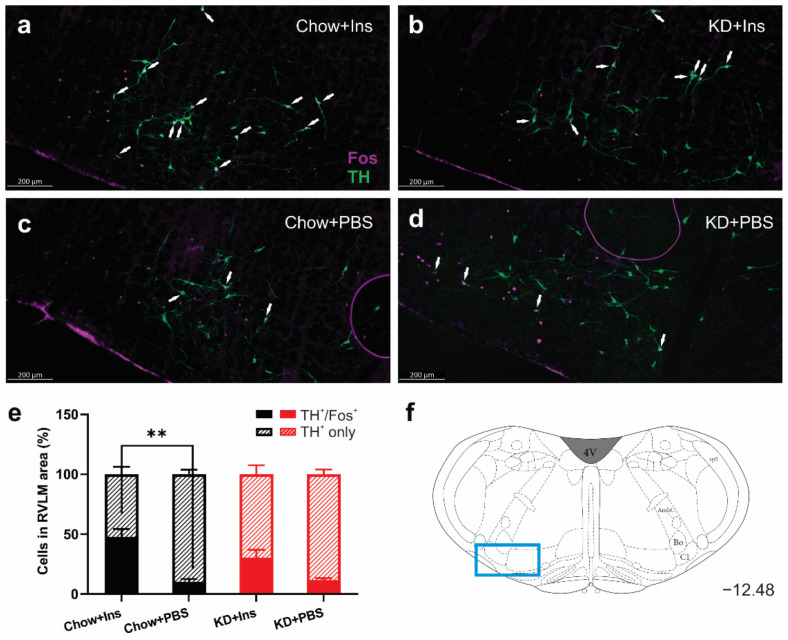
Insulin-induced hypoglycemia increases activation of medullary C1 neurons in Chow-fed animals. (**a**–**d**) Representative images of Fos (magenta) and TH (green) immunolabelling of C1 region of medulla oblongata in (**a**) Chow + Ins, (**b**) KD + Ins, (**c**) Chow + PBS, and (**d**) KD + PBS; white arrows indicate TH and Fos colocalization. (**e**) Grouped data showing the percentage of Fos-expressing TH^+^ C1 neurons (hatched bars TH^+^ only, solid bars TH^+^ and Fos^+^). (**f**) Representative images were taken at Bregma level −12.48 (blue rectangle). Data are means + SEM; ** *p* < 0.01; one-way ANOVA with Holm-Šidák’s test for multiple comparisons. Chow + Ins—chow-fed rats injected with insulin; KD + Ins—ketogenic diet-fed rats injected with insulin; Chow + PBS—chow-fed rats injected with vehicle PBS; KD + PBS—ketogenic diet fed rats injected with vehicle PBS.

The activation of tyrosine hydroxylase-expressing (TH) neurons in the ventrolateral medulla (C1 area) is necessary for stimulation of epinephrine secretion from the adrenal gland [35]. The number of TH-immunoreactive neurons in the RVLM did not differ be-tween treatments (Figure 6 a–d), whereas the proportion of activated (TH^+^/Fos^+^) neurons significantly increased in the Chow + Ins group (Figure 6a,e) compared to the corresponding PBS control (Figure 6a vs. Figure 6c,e). Although there was an increase in TH^+^/Fos^+^ in the KD + Ins group compared to KD + PBS (30.3% ± 6.6% vs. 11.7% ± 1.7%, respectively, Figure 6b,e), the difference was not statistically significant. These results demonstrate the involvement of the brainstem glucoregulatory nuclei in the CRR under both Chow and KD conditions. 

### 3.5. Chromaffin Cell Activity and Epinephrine Secretion Are Not Diminished in KD-Fed Rats

**Figure 7:**
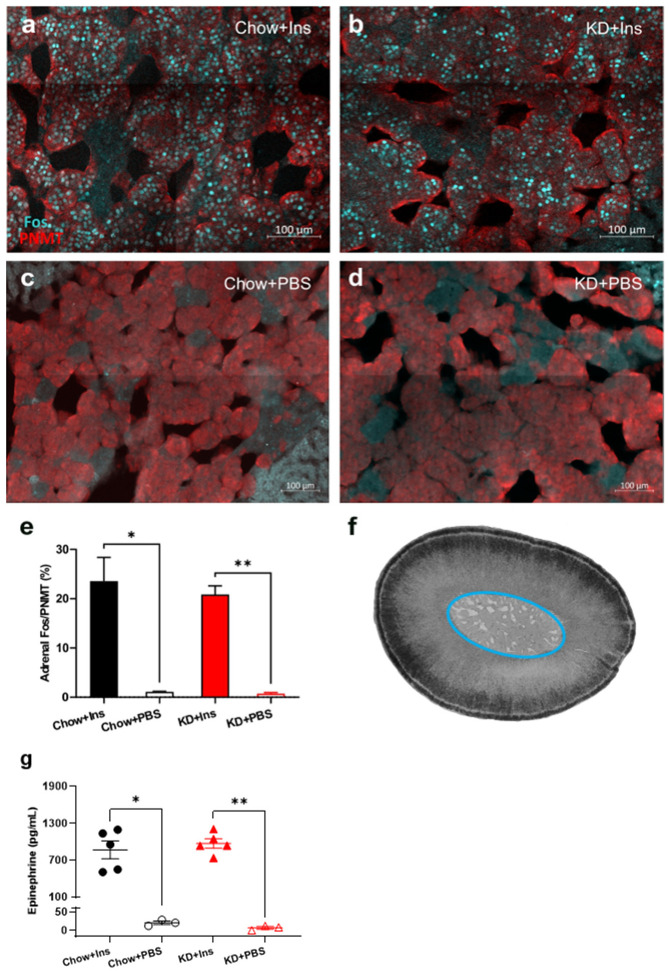
Ketogenic diet does not impair adrenal gland activity and epinephrine release. (**a**–**d**) Representative images of Fos (cyan) and PNMT (red) staining of adrenal medulla 2 h post insulin administration in (**a**) Chow + Ins and (**b**) KD + Ins rats or PBS administration in (**c**) Chow + PBS and (**d**) KD + PBS groups. (**e**) Grouped data showing the proportion of Fos-immunoreactive epinephrine-producing (PNMT-positive) chromaffin cells is significantly elevated following insulin injection in both Chow and KD-fed animals (*n* = 5 sections/group obtained from 3–5 animals). (**f**) Brightfield image of an adrenal gland section with the medulla circled in blue. (**g**) Plasma epinephrine levels were significantly higher in Chow + Ins (*n* = 5, black solid circles) and KD + Ins (*n* = 5, red solid triangles) rats compared to Chow + PBS (*n* = 3, black empty circles) and KD + PBS (*n* = 3, red empty triangles) rats, respectively. Data are means ± SEM; * *p* < 0.05, ** *p* < 0.01; Brown-Forsythe ANOVA with Dunnett’s test for multiple comparisons. Chow + Ins—chow-fed rats injected with insulin; KD + Ins—ketogenic diet-fed rats injected with insulin; Chow + PBS—chow-fed rats injected with vehicle PBS; KD + PBS—ketogenic diet fed rats injected with vehicle PBS.

The proportion of Fos-positive, PNMT-immunoreactive (epinephrine-producing) chromaffin cells in the adrenal medulla and plasma epinephrine levels were quantified 2 h after insulin administration to establish that hypoglycemia activated the sympathoadrenal CRR (Figure 7). PBS-treated (control) Chow- and KD-fed rats had virtually no Fos immunoreactivity (1.1% ± 0.1% and 0.7% ± 0.2%, respectively, Figure 7c–e) and correspondingly low levels of epinephrine (16.2 ± 5.8 pg/mL and 2.9 ± 7.7 pg/mL respectively, Figure 7g). Insulin-induced hypoglycemia significantly increased Fos immunoreactivity in both Chow-fed rats (23.6 ± 4.8%, *p* = 0.0001, Figure 7a,e) and KD-fed rats (20.9 ± 1.8%, *p* = 0.0007, Figure 7b,e). Epinephrine levels were significantly and similarly increased in Chow-fed and KD-fed rats (869.7 ± 144.5 pg/mL and 973.2 ± 77.1 pg/mL, respectively, Figure 7g) concurrently with adrenal chromaffin cell activation, suggesting that the neuroendocrine function of adrenal chromaffin cells is preserved in the KD-fed rats.

## 4. Discussion

It was previously reported that acute administration of ketones leads to attenuation of the counterregulatory response to insulin-induced hypoglycemia, but studies on the effects of chronic diet-induced ketosis are lacking. Here, we report that a 3 week KD in rats (1) induces stable ketosis, (2) does not impair the counterregulatory epinephrine release in response to severe insulin-induced hypoglycemia, (3) does not diminish the sympathoadrenal response as assessed by adrenal sympathetic nerve activity and adrenal gland function, and (4) shifts the onset of sympathoadrenal counterregulatory response to lower blood glucose levels. The delay in the sympathoadrenal response is concurrent with rapid cerebral and peripheral ketone consumption and diminished activation of low-glucose-sensitive C1 neurons in the brainstem. Our findings support the notion that ketones may be an effective alternative (or additional) energy source at times of decreased glucose availability; however, in contrast to other studies, nutritional ketosis does not decrease the magnitude of counterregulatory sympathoadrenal response.

Elevated levels of plasma ketone bodies are associated with increased cerebral uptake and utilization of ketones [13,36,37], as well as a parallel decrease in brain glucose metabolic rates [34], indicating a glucose-sparing effect and a metabolic switch toward ketone metabolism, possibly explaining the delayed ASNA response in KD-fed rats in the present study. The degree of cerebral ketone uptake and the glucose-sparing effect of ketones depend on the duration and magnitude of ketosis [34,38]. During prolonged fasting ketones can provide up to 60% of the brain’s energy requirements [14]; additionally, with every 1 mmol/L increase in plasma ketones, brain glucose utilization rate decreases by 9–10% [15,34,39]. In our study, a 3 week KD regimen was enough to raise blood BHB levels to 4.1 mmol/L, a level close to the *K*_m_ for brain endothelial ketone transport by MCT1 (monocarboxylate transporter 1) [39], suggesting that the concentration of BHB was sufficient to exert effects on the brain. In contrast, overnight fasting-induced elevation of BHB to ~1.6 mmol/L in Chow-fed rats was insufficient to optimally contribute to neuronal metabolism and probably did not play a significant role in the CRR to hypoglycemia. A sharp reduction in cerebral and peripheral BHB levels in the KD-fed rats was observed within 30 min of insulin administration, which coincided with a slow ASNA increase during this period. Once the fall in BHB plateaued at 30 min, ASNA increased exponentially. Given that insulin rapidly inhibits ketogenesis [40], the decreasing levels of BHB in both peripheral and cerebral blood are indicative of net BHB consumption by extrahepatic tissues, including the brain. 

While we did not directly measure brain tissue consumption of BHB, we can speculate on the basis of BHB measurements in the cerebral venous blood that central glucose-inhibited neurons (i.e., neurons that increase their firing rate in the presence of low glucose) were not sufficiently activated until a substantial amount of BHB disappeared from the circulation, which manifested as a delayed increase in ASNA and epinephrine release. One of the important brain nuclei necessary for the CRR and specifically for the sympathetically mediated epinephrine secretion is the C1 area in the rostral ventrolateral medulla [24,41]. In our study, a smaller proportion of activated C1 neurons in KD-fed rats may indicate a delayed response to hypoglycemia due to the time-course of Fos expression, which is reported to peak at 2 h post stimulus in the medulla [42]; however, in the absence of a longer time-course (e.g., 3 h post insulin), lower (but not delayed) activation of C1 neurons cannot be excluded.

In addition to the preserved ASNA, adrenal gland activity was not adversely affected in the KD-fed rats, as evidenced by high level of chromaffin cell activation and plasma epinephrine concentration measured at the end of the experiment. This suggests a good agreement between the function of the adrenal gland and ASNA, which is not always the case. Previous studies in mice [43] and rats [25] revealed that, under conditions of recurrent hypoglycemia, ASNA does not correlate with epinephrine release, suggesting impairment of the CRR at the level of the adrenal gland. 

The mechanism of action of ketones in various physiological and pathological states has been studied extensively, mostly by analyzing the effects of acutely administered exogenous ketones [44,45,46,47], which produce similar level of ketosis as the KD. However, Poff et al. cautioned against the extrapolation of findings from acute studies to chronic KD-induced conditions, as the metabolic state resulting from KD consumption, although similar, is not identical to the one resulting from exogenous ketone administration [48]. This indeed appears to be the case in studies investigating the CRR to hypoglycemia; previous human studies showed that acute infusion of exogenous ketones causes a reduction in epinephrine levels [11,49], which contrasts with our findings in KD-fed rats. This discrepancy might be explained by the stimulatory effects of acute ketone administration on cerebral blood flow [50], which is associated with significantly reduced perception of hypoglycemia symptoms and blunted sympathoadrenal response in healthy humans [51]. These findings suggest that attenuated CRR to hypoglycemia in the settings of acute administration of ketones, as opposed to KD-induced sustained ketosis, might be due to the direct effects of ketones on cerebral blood flow, the elevation of which allows a faster delivery of all energy substrates, including glucose, to the brain rather than preferential neuronal metabolism of ketones. In fact, it was shown that chronic diet-induced ketosis does not alter cerebral blood flow in rats but increases capillary density in the brain [52]. In order to maximize cerebral uptake and efficiency of ketone utilization as an energy substrate, a period of adaptation is required, as occurs with KDs [53,54] and extended fasting [55,56,57,58]. Adaptation enables the upregulation of MCTs and ketolytic enzyme expression in the brain [59,60], whereas acute administration of exogenous sources of ketones does not [37,61]. In the present study, 3 weeks of KD was sufficient to induce a stable level of ketosis (>2.5 mmol/L), which was not further augmented by an overnight fast (fed level of ketones 3.2 ± 0.2 mmol/L, unpublished pilot studies) and, therefore, may be considered a maximum nutritional level; these characteristics were previously described and defined as keto-adaptation in KD-fed Sprague-Dawley rats [33]. The observed glucose-sparing effect may provide a metabolic energy buffer for the glucose-sensing neurons in the hypothalamus and brainstem during the initial stages of hypoglycemia, thereby delaying autonomic neurogenic symptoms such as shakiness, hunger, palpitations, and sweating (or evidence of increased ASNA in anesthetized animals); once the buffering capacity of ketones is exceeded, glucose-inhibited neurons are activated and the CRR is rapidly initiated. Further studies are warranted to investigate whether there is sufficient time for the sympathoadrenal response to increase endogenous glucose production before the development of neuroglycopenia under conditions of nutritional ketosis. 

The effect of the KD on blood pressure was not a focus of the current study; however, it is worth noting the significantly elevated baseline level of mean arterial pressure in the KD-fed rats. Although the exact mechanisms of the development of this observed increase in blood pressure is unknown, one possibility is the BHB-induced increase in sympathetic nerve activity. Indeed, one previous study did report a stimulatory effect of BHB added to the rat chow for 4 days on sympathetic nerve activity in rats [62]; however, the existing literature in this area is limited, particularly in animals [63]. It is well established that chronically elevated sympathetic nerve activity can lead to hypertension [64]; taken together, we can hypothesize that in the present study the elevated baseline blood pressure may have resulted from sympathoexcitatory effects of KD-induced chronic ketosis. However, without further investigation, other mechanisms cannot be ruled out. 

### Technical Considerations

This study has technical and interpretive limitations that must be considered. First, the invasive ASNA recordings reported in this study were only achievable in anesthetized animals and, as such, limited us to recording only severe symptoms of hypoglycemia, such as clonic convulsions in some Chow-fed animals. However, the measurements of physical symptoms of hypoglycemia were not our primary objective. One of the advantages of using an anesthetized preparation is that it eliminates handling stress-induced changes in epinephrine, one of the key measures of the CRR in our study. Second, it can be argued the measurements of epinephrine release in response to hypoglycemia are sufficient to assess the function of the sympathoadrenal system as it positively correlates with ASNA [27]; however, it is not always the case. As such, Sivitz et al. demonstrated that repeated exposure to insulin-induced hypoglycemia significantly reduces plasma epinephrine without reducing the level of ASNA [25]. Further studies by Ma et al. confirmed that the dysregulation of sympathoadrenal counterregulatory response originates at the level of adrenal gland, leading to impaired epinephrine release [43]. Therefore, it was prudent in the current study to assess multiple components of the sympathoadrenal response to pinpoint the possible source of impairment. Advantageously, ASNA recordings give a direct real-time measure of the sympathetic output following a hypoglycemic stimulus. This is important because, in our study, the precipitous decline in ASNA after an initial hypoglycemia-induced rise was predictive of the occurrence of clonic convulsions in Chow-fed rats (Table S1), a finding not possible with epinephrine measurement after termination of the experiment. Although not explored in the current study, this observation deserves further examination. Third, this study was focused primarily on the effects of circulating ketones on the CRR; however, the KD used here contained 34% of medium-chain fatty acids (from Copha—hydrogenated coconut oil), which are not only readily metabolized into ketone bodies in the liver but can also cross the blood-brain barrier (unlike long-chain fatty acids) and directly affect brain energy metabolism through various mechanisms [65]. Our experiments were not designed to assess the relative contribution of medium-chain fatty acids on the CRR, but their contribution to the demonstrated response cannot be excluded. Lastly, the study was conducted in healthy, nondiabetic rodents and, although it does provide novel insights into the normal hypoglycemia-induced CRR under the conditions of chronic nutritional ketosis, it would be optimal, albeit challenging, to study this response in animal models of diabetes.

## 5. Conclusions

The current study provides a novel insight into the sympathoadrenal CRR to insulin-induced hypoglycemia under conditions of nutritional ketosis in healthy, nondiabetic rats. We demonstrated that the sympathoadrenal response in KD-fed rats is as strong as in Chow-fed rats; however, it occurs at lower blood glucose levels. Were similar effects to occur in type 1 diabetes, KD might be a useful strategy to mitigate symptoms and consequences of hypoglycemia while providing the brain with an additional metabolic substrate.

## Data Availability

The original data used to support the findings of this study are available from the corresponding author upon request.

## Supplementary Materials

The following are available online at https://www.mdpi.com/article/10.3390/nu13082627/s1, Figure S1: Representative image showing a rat dorsal skull craniotomy exposing the confluence of sinuses, Figure S2: The effects of insulin-induced hypoglycemia on blood pressure and heart rate in anesthetized rats, Table S1. Grouped data for animals excluded due to clonic convulsions.

## References

[1] Geller A.I., Shehab N., Lovegrove M.C., Kegler S.R., Weidenbach K.N., Ryan G.J., Budnitz D. (2014). National Estimates of Insulin-Related Hypoglycemia and Errors Leading to Emergency Department Visits and Hospitalizations. JAMA Intern. Med..

[2] Skrivarhaug T., Bangstad H.-J., Stene L.C., Sandvik L., Hanssen K.F., Joner G. (2006). Long-term mortality in a nationwide cohort of childhood-onset type 1 diabetic patients in Norway. Diabetologia.

[3] Cryer P.E. (1993). Glucose counterregulation: Prevention and correction of hypoglycemia in humans. Am. J. Physiol. Metab..

[4] Tesfaye N., Seaquist E.R. (2010). Neuroendocrine responses to hypoglycemia. Ann. N. Y. Acad. Sci..

[5] Ritter S., Li A.-J., Wang Q., Dinh T.T. (2011). Minireview: The Value of Looking Backward: The Essential Role of the Hindbrain in Counterregulatory Responses to Glucose Deficit. Endocrinology.

[6] Rickels M.R. (2019). Hypoglycemia-associated autonomic failure, counterregulatory responses, and therapeutic options in type 1 diabetes. Ann. N. Y. Acad. Sci..

[7] Stanley S., Moheet A., Seaquist E.R. (2019). Central Mechanisms of Glucose Sensing and Counterregulation in Defense of Hypoglycemia. Endocr. Rev..

[8] Beall C., Ashford M., McCrimmon R.J. (2012). The physiology and pathophysiology of the neural control of the counterregulatory response. Am. J. Physiol. Integr. Comp. Physiol..

[9] Owen O.E., Morgan A.P., Kemp H.G., Sullivan J.M., Herrera M.G., Cahill G.F. (1967). Brain Metabolism during Fasting. J. Clin. Investig..

[10] Page K.A., Williamson A., Yu N., McNay E.C., Dzuira J., McCrimmon R.J., Sherwin R.S. (2009). Medium-Chain Fatty Acids Improve Cognitive Function in Intensively Treated Type 1 Diabetic Patients and Support in Vitro Synaptic Transmission during Acute Hypoglycemia. Diabetes.

[11] Veneman T., Mitrakou A., Mokan M., Cryer P., Gerich J. (1994). Effect of hyperketonemia and hyperlacticacidemia on symptoms, cognitive dysfunction, and counterregulatory hormone responses during hypoglycemia in normal humans. Diabetes.

[12] Yamada K.A., Rensing N., Thio L.L. (2005). Ketogenic diet reduces hypoglycemia-induced neuronal death in young rats. Neurosci. Lett..

[13] Drenick E.J., Alvarez L.C., Tamasi G.C., Brickman A.S. (1972). Resistance to Symptomatic Insulin Reactions after Fasting. J. Clin. Investig..

[14] Cahill G.F. (2006). Fuel Metabolism in Starvation. Annu. Rev. Nutr..

[15] LaManna J.C., Salem N., Puchowicz M., Erokwu B., Koppaka S., Flask C., Lee Z. (2009). Ketones Suppress Brain Glucose Consumption. Adv. Exp. Med. Biol..

[16] Kossoff E., Wang H.-S., Eh K., Hs W. (2013). Dietary Therapies for Epilepsy. Biomed. J..

[17] Lennerz B.S., Barton A., Bernstein R.K., Dikeman R.D., Diulus C., Hallberg S., Rhodes E.T., Ebbeling C.B., Westman E.C., Yancy W.S. (2018). Management of Type 1 Diabetes With a Very Low–Carbohydrate Diet. Pediatrics.

[18] Leow Z.Z.X., Guelfi K., Davis E.A., Jones T.W., Fournier P.A. (2018). The glycaemic benefits of a very-low-carbohydrate ketogenic diet in adults with Type 1 diabetes mellitus may be opposed by increased hypoglycaemia risk and dyslipidaemia. Diabet. Med..

[19] Nolan J., Rush A., Kaye J. (2019). Glycaemic stability of a cyclist with Type 1 diabetes: 4011 km in 20 days on a ketogenic diet. Diabet. Med..

[20] Frølund L., Kehlet H., Christensen N.J., Alberti K.G.M. (1980). Effect of ketone body infusion on plasma catecholamine and substrate concentrations during acute hypoglycemia in man. J. Clin. Endocrinol. Metab..

[21] Morrison C.D., Hill C.M., Duvall M.A., Coulter C.E., Gosey J.L., Herrera M.J., Maisano L.E., Sikaffy H.X., McDougal D.H. (2020). Consuming a ketogenic diet leads to altered hypoglycemiccounter-regulation in mice. J. Diabetes Complicat..

[22] Ranjan A., Schmidt S., Damm-Frydenberg C., Steineck I., Clausen T.R., Holst J.J., Madsbad S., Norgaard K. (2017). Low-Carbohydrate Diet Impairs the Effect of Glucagon in the Treatment of Insulin-Induced Mild Hypoglycemia: A Randomized Crossover Study. Diabetes Care.

[23] Senthilkumaran M., Bobrovskaya L. (2018). The effects of recurrent hypoglycaemia and opioid antagonists on the adrenal catecholamine synthetic capacity in a rat model of HAAF. Auton. Neurosci..

[24] Kakall Z.M., Kavurma M.M., Cohen E.M., Howe P.R., Nedoboy P.E., Pilowsky P.M. (2019). Repetitive hypoglycemia reduces activation of glucose-responsive neurons in C1 and C3 medullary brain regions to subsequent hypoglycemia. Am. J. Physiol. Metab..

[25] Sivitz W.I., Herlein J.A., Morgan N.A., Fink B.D., Phillips B.G., Haynes W.G. (2001). Effect of acute and antecedent hypoglycemia on sympathetic neural activity and catecholamine responsiveness in normal rats. Diabetes.

[26] Kakall Z.M., Nedoboy P., Farnham M.M.J., Pilowsky P.M. (2018). Activation of µ-opioid receptors in the rostral ventrolateral medulla blocks the sympathetic counterregulatory response to glucoprivation. Am. J. Physiol. Integr. Comp. Physiol..

[27] Korim W.S., Farah L.B., McMullan S., Verberne A. (2014). Orexinergic Activation of Medullary Premotor Neurons Modulates the Adrenal Sympathoexcitation to Hypothalamic Glucoprivation. Diabetes.

[28] Sano Y., Ito S., Yoneda M., Nagasawa K., Matsuura N., Yamada Y., Uchinaka A., Bando Y.K., Murohara T., Nagata K. (2016). Effects of various types of anesthesia on hemodynamics, cardiac function, and glucose and lipid metabolism in rats. Am. J. Physiol. Circ. Physiol..

[29] Rocha M., Herbert H. (1997). Effects of anesthetics on Fos protein expression in autonomic brain nuclei related to cardiovascular regulation. Neuropharmacology.

[30] Livett B., Day R., Elde R., Howe P. (1982). Co-storage of enkephalins and adrenaline in the bovine adrenal medulla. Neuroscience.

[31] Howe P., Costa M., Furness J.B., Chalmers J. (1980). Simultaneous demonstration of phenylethanolamine N-methyltransferase immunofluorescent and catecholamine fluorescent nerve cell bodies in the rat medulla oblongata. Neuroscience.

[32] Nedoboy P., Mohammed S., Kapoor K., Bhandare A., Farnham M., Pilowsky P. (2016). pSer40 tyrosine hydroxylase immunohistochemistry identifies the anatomical location of C1 neurons in rat RVLM that are activated by hypotension. Neuroscience.

[33] Hernandez A., Truckenbrod L., Federico Q., Campos K., Moon B., Ferekides N., Hoppe M., D’Agostino D., Burke S. (2020). Metabolic switching is impaired by aging and facilitated by ketosis independent of glycogen. Aging.

[34] Zhang Y., Kuang Y., Xu K., Harris D., Lee Z., LaManna J., A Puchowicz M. (2013). Ketosis Proportionately Spares Glucose Utilization in Brain. Br. J. Pharmacol..

[35] Ritter S., Bugarith K., Dinh T.T. (2001). Immunotoxic destruction of distinct catecholamine subgroups produces selective impairment of glucoregulatory responses and neuronal activation. J. Comp. Neurol..

[36] Nehlig A. (2004). Brain uptake and metabolism of ketone bodies in animal models. Prostaglandins Leukot. Essent. Fat. Acids.

[37] Hasselbalch S.G., Madsen P.L., Hageman L.P., Olsen K.S., Justesen N., Holm S., Paulson O.B. (1996). Changes in cerebral blood flow and carbohydrate metabolism during acute hyperketonemia. Am. J. Physiol. Metab..

[38] Morris A.A.M. (2005). Cerebral ketone body metabolism. J. Inherit. Metab. Dis..

[39] Veech R.L. (2004). The therapeutic implications of ketone bodies: The effects of ketone bodies in pathological conditions: Ketosis, ketogenic diet, redox states, insulin resistance, and mitochondrial metabolism. Prostaglandins Leukot. Essent. Fat. Acids.

[40] DeFronzo R.A., Ferrannini E. (2016). Regulation of Intermediary Metabolism during Fasting and Feeding. Endocrinology: Adult and Pediatric.

[41] Li A.-J., Wang Q., Ritter S. (2018). Selective Pharmacogenetic Activation of Catecholamine Subgroups in the Ventrolateral Medulla Elicits Key Glucoregulatory Responses. Endocrinology.

[42] Chan R.K., Sawchenko P.E. (1994). Spatially and temporally differentiated patterns of c-fos expression in brainstem catecholaminergic cell groups induced by cardiovascular challenges in the rat. J. Comp. Neurol..

[43] Ma Y., Wang Q., Joe D., Wang M., Whim M.D. (2018). Recurrent hypoglycemia inhibits the counterregulatory response by suppressing adrenal activity. J. Clin. Investig..

[44] Haces M.L., Hernández-Fonseca K., Medina-Campos O.N., Montiel T., Pedraza-Chaverri J., Massieu L. (2008). Antioxidant capacity contributes to protection of ketone bodies against oxidative damage induced during hypoglycemic conditions. Exp. Neurol..

[45] Julio-Amilpas A., Montiel T., Soto-Tinoco E., Gerónimo-Olvera C., Massieu L. (2015). Protection of hypoglycemia-induced neuronal death by beta-hydroxybutyrate involves the preservation of energy levels and decreased production of reactive oxygen species. J. Cereb. Blood Flow Metab..

[46] Gormsen L.C., Svart M., Thomsen H.H., Søndergaard E., Vendelbo M.H., Christensen N., Tolbod L.P., Harms H.J., Nielsen R., Wiggers H. (2017). Ketone Body Infusion With 3-Hydroxybutyrate Reduces Myocardial Glucose Uptake and Increases Blood Flow in Humans: A Positron Emission Tomography Study. J. Am. Heart Assoc..

[47] Kovacs Z., D’Agostino D.P., Diamond D., Kindy M.S., Rogers C., Ari C. (2019). Therapeutic Potential of Exogenous Ketone Supplement Induced Ketosis in the Treatment of Psychiatric Disorders: Review of Current Literature. Front. Psychiatry.

[48] Poff A.M., Koutnik A.P., Egan B. (2020). Nutritional Ketosis with Ketogenic Diets or Exogenous Ketones: Features, Convergence, and Divergence. Curr. Sports Med. Rep..

[49] Amiel S.A., Archibald H.R., Chusney G., Williams A.J.K., Gale E.A.M. (1991). Ketone infusion lowers hormonal responses to hypoglycaemia: Evidence for acute cerebral utilization of a non-glucose fuel. Clin. Sci..

[50] Linde R., Hasselbalch S.G., Topp S., Paulson O.B., Madsen P.L. (2006). Global cerebral blood flow and metabolism during acute hyperketonemia in the awake and anesthetized rat. J. Cereb. Blood Flow Metab..

[51] Thomas M., Sherwin R.S., Murphy J., Kerr D. (1997). Importance of cerebral blood flow to the recognition of and physiological responses to hypoglycemia. Diabetes.

[52] Puchowicz M.A., Xu K., Sun X., Ivy A., Emancipator D., Lamanna J.C. (2007). Diet-induced ketosis increases capillary density without altered blood flow in rat brain. Am. J. Physiol. Metab..

[53] Leino R.L., Gerhart D.Z., Duelli R., Enerson B.E., Drewes L.R. (2001). Diet-induced ketosis increases monocarboxylate transporter (MCT1) levels in rat brain. Neurochem. Int..

[54] Pierre K., Parent A., Jayet P.-Y., Halestrap A.P., Scherrer U., Pellerin L. (2007). Enhanced expression of three monocarboxylate transporter isoforms in the brain of obese mice. J. Physiol..

[55] Pan J.W., Rothman D.L., Behar K.L., Stein D.T., Hetherington H.P. (2000). Human brain beta-hydroxybutyrate and lactate increase in fasting-induced ketosis. J. Cereb. Blood Flow Metab..

[56] Hasselbalch S.G., Knudsen G.M., Jakobsen J., Hageman L.P., Holm S., Paulson O.B. (1995). Blood-brain barrier permeability of glucose and ketone bodies during short-term starvation in humans. Am. J. Physiol. Metab..

[57] Matsuyama S., Ohkura S., Iwata K., Uenoyama Y., Tsukamura H., Maeda K.-I., Kimura K. (2009). Food Deprivation Induces Monocarboxylate Transporter 2 Expression in the Brainstem of Female Rat. J. Reprod. Dev..

[58] Pitchaimani V., Arumugam S., Thandavarayan R.A., Gounder V.K., Afrin M.R., Sreedhar R., Harima M., Suzuki H., Miyashita S., Nakamura T. (2016). Fasting time duration modulates the onset of insulin-induced hypoglycemic seizures in mice. Epilepsy Res..

[59] Prins M.L. (2008). Cerebral Metabolic Adaptation and Ketone Metabolism after Brain Injury. Br. J. Pharmacol..

[60] Amiel S.A. (1997). Hypoglycaemia in diabetes mellitus--protecting the brain. Diabetologia.

[61] Pan J.W., Telang F.W., Lee J.H., De Graaf R.A., Rothman D.L., Stein D.T., Hetherington H.P. (2001). Measurement of beta-hydroxybutyrate in acute hyperketonemia in human brain. J. Neurochem..

[62] Kolanowski J., Young J.B., Landsberg L. (1994). Stimulatory influence of d(−)3-hydroxybutyrate feeding on sympathetic nervous system activity in the rat. Metabolism.

[63] Kosinski C., Jornayvaz F.R. (2017). Effects of Ketogenic Diets on Cardiovascular Risk Factors: Evidence from Animal and Human Studies. Nutrients.

[64] Grassi G., Mark A., Esler M. (2015). The Sympathetic Nervous System Alterations in Human Hypertension. Circ. Res..

[65] Augustin K., Khabbush A., Williams S., Eaton S., Orford M., Cross H., Heales S.J.R., Walker M.C., Williams R.S.B. (2018). Mechanisms of action for the medium-chain triglyceride ketogenic diet in neurological and metabolic disorders. Lancet Neurol..

