# Supplementary material for: Slow but Steady—The Responsiveness of Sympathoadrenal System to a Hypoglycemic Challenge in Ketogenic Diet-Fed Rats
Source: Nutrients. 2021 Jul 29;13(8):2627. doi: 10.3390/nu13082627 (PMC8398867; doi:10.3390/nu13082627)
Supplement: Supplementary file 1 [file nutrients-13-02627-s001.zip › nutrients-1308870-supplementary.pdf]

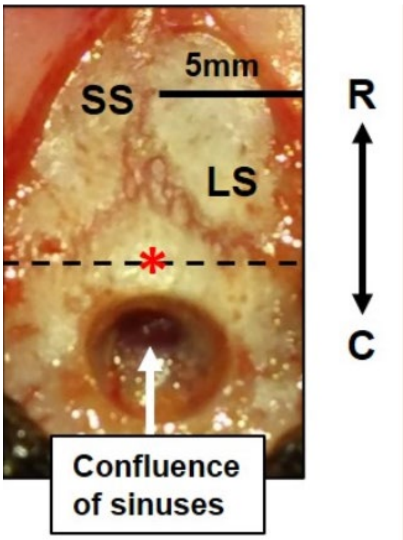

Supplementary Figure S1. Representative image showing a rat dorsal skull craniotomy exposing the confluence of sinuses. C – caudal, R – rostral, LS – lambdoid suture, SS – sagittal suture, dashed line – interaural line, red asterisk – Lambda.

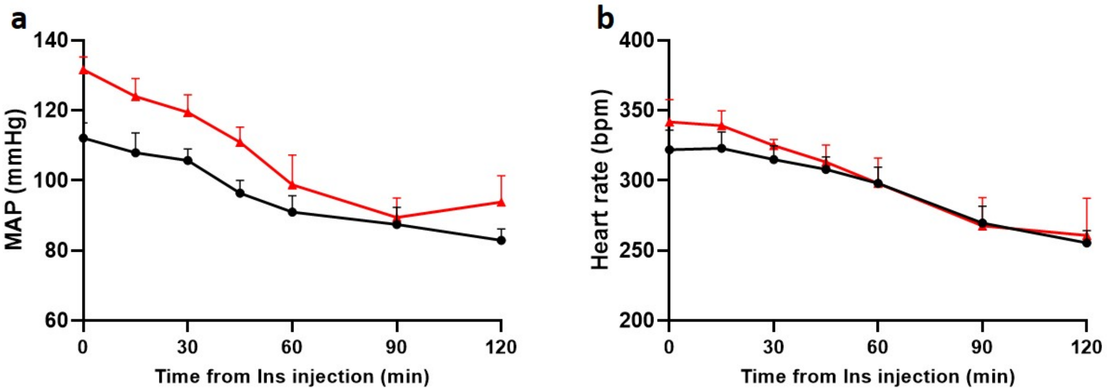

Supplementary Figure S2. The effects of insulin-induced hypoglycemia on blood pressure and heart rate in anesthetized rats. (**a**), Mean arterial pressure (MAP) and (**b**), Heart rate progressively decreased during the 2-h recording period in all groups; CHOW+Ins – black line, KD+Ins – red line. Data are mean + SEM

Supplementary Table S1. Grouped data for animals excluded due to clonic convulsions.

Time post Ins injection	0min (n=4)	15min (n=4)	30min (n=4)	45min (n=4)	60min (n=4)	90min (n=4)		120min (n=2)	
ASNA (%Δ from baseline)	0	23.1 ± 14.4	60.8 ± 18.2	106.8 ± 30.5	90.3 ± 26.7	68.2 ± 38.2	Convulsions (n=2)	-2.1 ± 4.4	Convulsions (n=2)
Blood glucose (mmol/L)	6.2 ± 0.7	4.7 ± 0.5	3.6 ± 0.4	2.9 ± 0.4	2.4 ± 0.3	1.8 ± 0.2		1.7 ± 0.6	
Blood BHB (mmol/L)	1.6 ± 0.2	1.4 ± 0.4	0.8 ± 0.1	0.8 ± 0.2	0.7 ± 0.1	0.5 ± 0.1		0.7 ± 0.2	

Values are mean ± SEM, number of animals are in parentheses.
